# Validation of an equine serum amyloid A assay with an unusually broad working range

**DOI:** 10.1186/s12917-019-2211-3

**Published:** 2019-12-19

**Authors:** Stine Jacobsen, Anne Mette Vinther, Mads Kjelgaard-Hansen, Lise Nikolic Nielsen

**Affiliations:** 1 Department of Veterinary Clinical Sciences, Section of Medicine and Surgery, University of Copenhagen, Agrovej 8, Taastrup, Denmark; 2 Ascendis Pharma A/S, Tuborg Boulevard 12, Hellerup, Denmark; 3 Department of Veterinary Clinical Sciences, The Veterinary Diagnostic Laboratory, University of Copenhagen, Dyrlaegevej 46, Frederiksberg C, Denmark

**Keywords:** Assay, Horse, Inflammation, Serum amyloid A, Validation

## Abstract

**Background:**

Serum amyloid A (SAA) is a major equine acute phase protein and of great value in detection and monitoring of inflammation. A new immunoturbidometric assay based on monoclonal antibodies (VET-SAA, Eiken Chemical Co., Japan) may be useful for SAA measurements in routine diagnostic laboratories. The aim of the study was to validate the VET-SAA immunoturbidometric assay and use it to measure serum SAA concentrations in a variety of clinical cases. Precision was assessed by intra- and interassay coefficients of variation of repeated measurements of serum pools (low, intermediate, high concentrations of SAA). Accuracy was estimated by linearity under dilution. Detection limit was determined by replicate determinations of ionized water. Measurements were compared to measurements performed in a previously validated SAA assay (LZSAA assay, Eiken Chemical Co., Japan). Subsequently, the VET-SAA assay was used for measuring serum SAA concentrations in horses with and without inflammation.

**Results:**

Detection limit was 1.2 mg/L. Without modifications, the assay measured SAA concentrations with acceptable reliability in a broad concentration range (0 to > 6000 mg/L). In the 0–3000 mg/L range, the assay demonstrated good precision and accuracy, and concentrations correlated well with those obtained in the LZSAA assay, albeit with a slight systematic bias. Concentrations of SAA assessed in horses with and without inflammation followed the expected pattern, with significantly higher concentrations in horses with systemic inflammation than in healthy horses and horses with non-inflammatory disease.

**Conclusions:**

The assay was unique in its ability to measure SAA concentrations with acceptable reliability over an extreme concentration range. This is relevant in the equine species, where SAA concentrations may reach very high concentrations.

## Background

Serum amyloid A (SAA) has been established as a sensitive and highly relevant marker of inflammation in horses [1–3]. SAA is a major acute phase protein in the horse, with this species seemingly unique in its ability to produce vast amounts of SAA in response to an inflammatory stimulus. Concentrations range from essentially 0 mg/L in healthy individuals to several thousand mg/L in horses with severe inflammation [4]. The consistently low concentrations found in healthy horses and the extreme magnitude of response renders SAA a particularly useful marker of inflammation, as shown in a variety of clinical conditions [5–10].

Assays relevant for assessment of SAA horse-side or in the smaller laboratories of general practitioners have been validated [11, 12]. These appear reliable, but will often measure SAA in a limited concentration range, e.g. up to 3000 mg/L. To be able to gauge severity/extent of inflammation, and to be able to monitor changes in inflammatory activity in severely ill horses, the ideal SAA assay must be able to measure concentrations reliably in an extremely broad range of concentrations without the need for multiple manual dilutions, as this increases reagent and labour costs and potentially jeopardizes the reliability of the assay. In our hospital, we have acquired extensive knowledge on the equine SAA response by measuring SAA in every horse admitted since 2007 using a previously validated immunoturbidometric assay (LZ SAA, Eiken Chemical Co., Tokyo, Japan) [13]. While this assay has proven reliable, it has two drawbacks: it is based on a combination of monoclonal and polyclonal antibodies, and it is linear in a limited concentration range. This means that 1) there is a higher potential for batch to batch variation due to the polyclonal antibodies [14, 15], and 2) samples from horses with severe inflammation need to be extensively diluted and go through repeated analysis to obtain a final SAA concentration. SAA concentrations of 3000–5000 mg/L are not uncommon, and in horses with severe and extensive inflammation (e.g. horses with peritonitis, colitis or lymphangitis), SAA concentrations may reach levels of 12.000–15.000 mg/L (unpublished data). To achieve absolute SAA concentrations, we have thus set the LZ SAA assay up with a 1: 5 reflex dilution of samples containing SAA concentrations > 300 mg/L [13] and with further manual dilutions of the sample, where this reflex dilution is insufficient.

Although the LZ SAA assay has performed reliably in our setting to date, an assay based purely on monoclonal antibody would be preferable to increase specificity and inter-batch consistency. Furthermore, there is a need for an assay that measures equine SAA concentration with good reliability in the extreme concentration range encountered in horses. The purpose of the present study was thus to validate a new assay for detection of SAA in the horse, which has been developed to address these issues.

## Results

### Assay characteristics

**Fig. 1:**
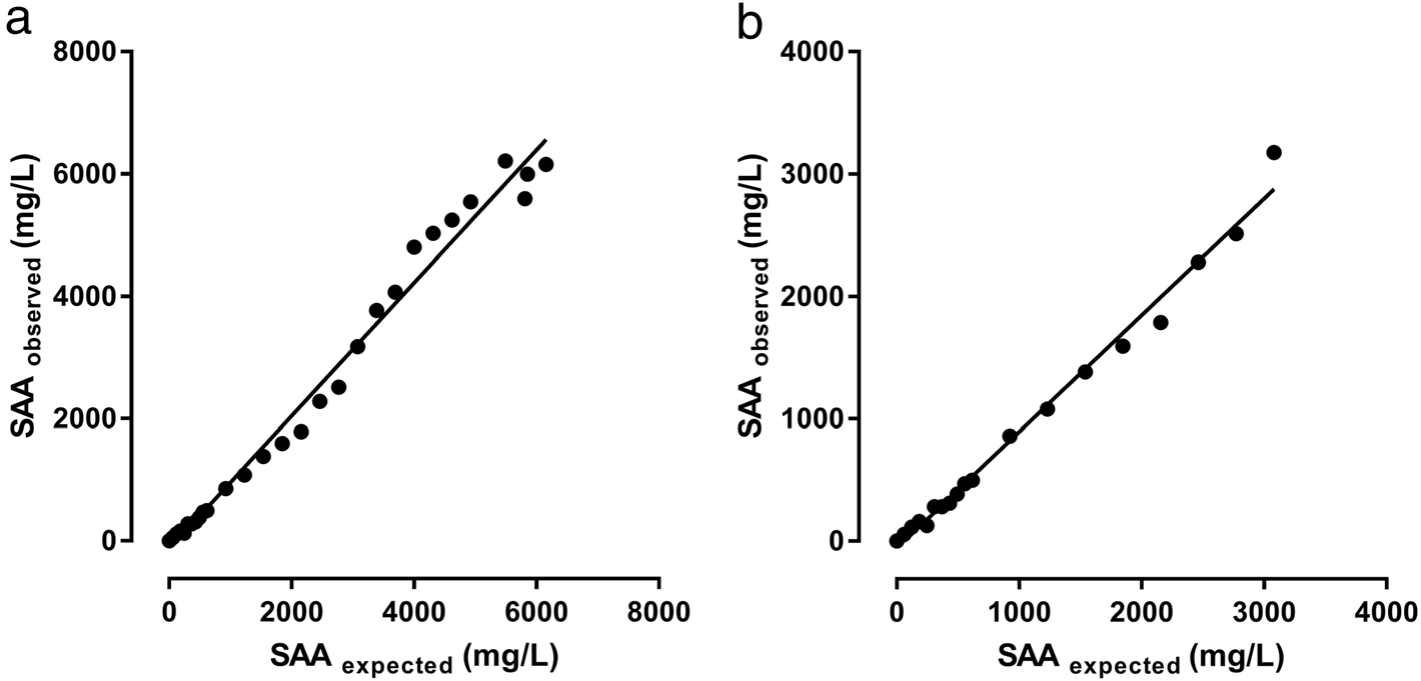
Linearity under dilution of an equine serum pool with high concentration of serum amyloid A (SAA). Determinations were performed by means of an immunotubidometric assay developed for determination of SAA concentrations in horses (VET-SAA). Panel **a** shows linearity under dilution in the full concentrations range, panel **b** shows linearity under dilution in the most frequently encountered/relevant concentration range (0–3000 mg/L). While the assay showed slight signs of inaccuracy in the full concentration range (**a**, Table 2), no significant deviation from a slope equal to 1 and a y-intercept equal to 0 were observed in the linear regression equation (x = the expected level according to dilution, y = the observed concentration) in the 0 to approximately 3000 mg/L concentration range (**b**, Table 2)

Intra- and inter-assay coefficients of variation (CVs) ranged from 3.0 to 5.2% and 6.8 to 9.6%, respectively (Table 1). Statistically significant deviations from a slope equal to 1 and a y-intercept equal to 0 were observed in the linear regression equation of the full concentration range of the diluted pool (Fig. 1a; Table 2), and Runs test revealed that data deviated from the linear model (*P* = 0.0002; Table 2). When linearity under dilution was assessed in the 0 to approximately 3000 mg/L concentration range, no signs of inaccuracy were observed (Fig. 1b; Table 2).

**Table 1:** Intra- and inter-assay variation in determination of serum amyloid (SAA) concentrations in equine serum samples

Comparison	No. of repeats	SAA concentration (mg/L)		Coefficient of variation (%)
		Mean	SD	
Intra-assay				
Low conc.	10	124.7	3.8	3.0
Intermediate conc.	10	805.3	41.9	5.2
High conc.	10	4443	161.3	3.6
Inter-assay				
Low conc.	10	113.4	7.7	6.8
Intermediate conc.	10	842.0	63.6	7.6
High conc.	10	4646	445.7	9.6

**Table 2:** Analytical inaccuracy of serum amyloid A (SAA) concentrations assessed by dilution of an equine serum pool

Full concentrations range (up to 6159.7 mg/L)	y-intercept (95% confidence interval)	− 130 (−293.3, 33.19)
	Slope (95% confidence interval)	1.087 (1.035, 1.140)
	P (Runs test)	0.0002
	r^2^	0.99
Concentration range up to 3000 mg/L	y-intercept (95% confidence interval)	− 58.5(− 129.6, 12.59)
	Slope (95% confidence interval)	0.952 (0.902, 1.003)
	P (Runs test)	0.4
	r^2^	0.99

The detection limit (DL) of the VET-SAA assay was 1.16 mg/L (blank measurements mean 0.77 mg/L; standard deviation [SD] 0.13 mg/L).

The bias percentages were within the predefined acceptance criterion for both interfering substances (Additional file 1).

### Method comparisons and overlap performance

**Fig. 2:**
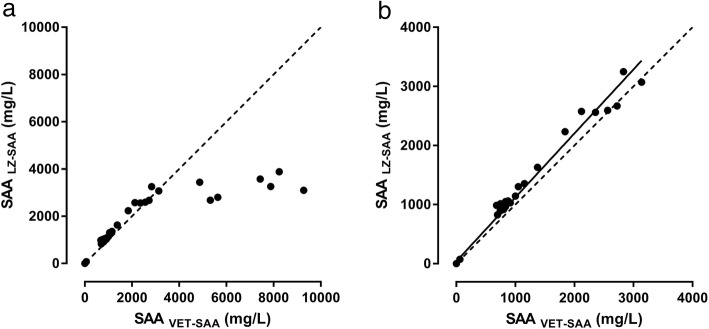
Comparison of serum amyloid A (SAA) concentrations measured in a novel immunotubidometric assay developed for determination of SAA concentrations in horses (VET-SAA) and in an immunotubidometric assay previously validated for use in horses (LZ SAA). Panel (**a**) shows method comparison in the full concentration range, panel (**b**) shows method comparison in the most frequently encountered/relevant concentration range (0–3000 mg/L), solid line in (**b**) = Deming regression line, dashed lines = perfect agreement (X = Y). Concentrations assessed in the LZ SAA assay trailed off at around 3000 mg/L causing poor correlation between the two assays at higher concentrations (**a**), but in the 0 to approximately 3000 mg/L concentration range, a high degree of correlation was observed between the two assays (**b**)

**Fig. 3:**
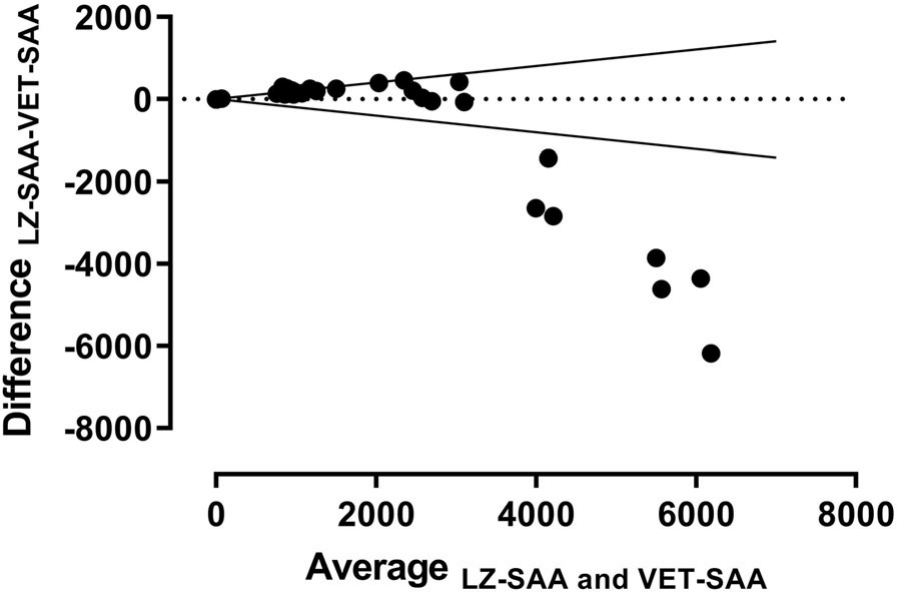
Bland-Altman plot of serum amyloid A (SAA) concentrations measured in a novel immunotubidometric assay developed for determination of SAA concentrations in horses (VET-SAA) and in an immunotubidometric assay previously validated for use in horses (LZ SAA). The difference between concentration readout in the two assays (y-axis) is plotted against the average SAA concentration (x-axis). The plot shows a large proportional and systematic bias, where the negative difference between concentrations measured in the two assays increases with increasing SAA concentration. The bias clearly exceeds the limits of the inherent combined imprecision of the assays (full lines), when concentrations exceeds 4000 mg/L (Data on LZ SAA imprecision derived from [13]; limits calculated as described in [16])

When the two assays were compared in the 0 to approximately 3000 mg/L concentration range, a high degree of correlation was observed between them (Fig. 2b;). The Deming regression of method comparison revealed a small proportional disagreement (slope [95% confidence interval] was 1.08 [1.04, 1.12]), but no systematic inaccuracy (Y-intercept [95% confidence interval] was 39.6 [− 7.6, 86.8]). The proportional disagreement was confirmed by direct plot of SAA measurements obtained by the two assays (Fig. 2b), visualizing the tendency of the LZSAA to result in slightly (8%) higher concentrations of SAA than the VET-SAA. When the two assays were compared in the full concentration range (0 to 9274 mg/L), there was large proportional and systematic bias (Fig. 3), caused by concentrations assessed in the LZSAA assay trailing off at concentrations higher than 3000 mg/L (Fig. 2a).

**Fig. 4:**
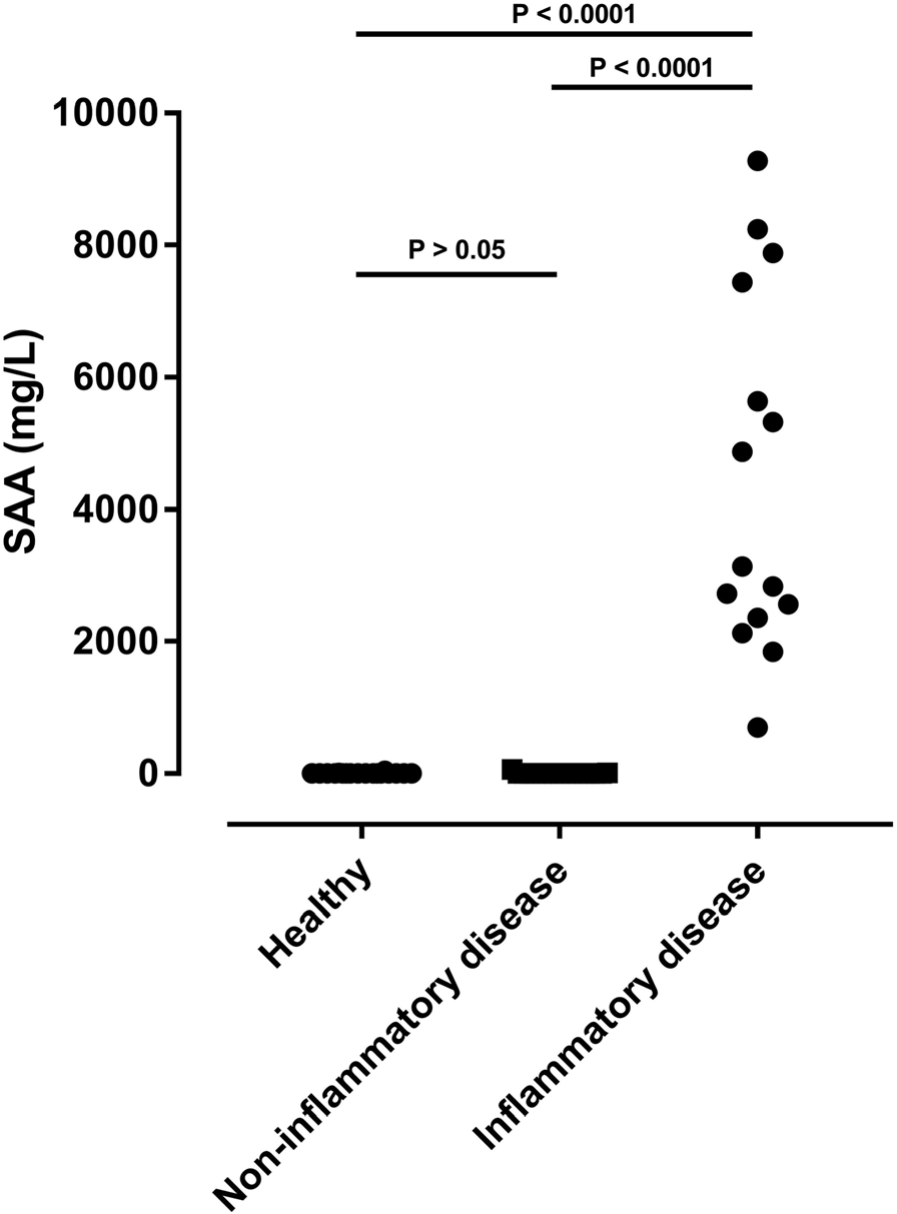
Serum concentrations of serum amyloid A (SAA) determined in different groups of horses by means of an immunotubidometric assay developed for determination of SAA concentrations in horses (VET-SAA)

Overlap assessment revealed the expected differences between healthy horses and horses with non-inflammatory and inflammatory disease. Median concentration in the three groups were 4.3, 6.1, and 4461 mg/L (Fig. 4). Concentrations of SAA in the three groups differed significantly (*P* < 0.0001), with levels in horses with inflammation being significantly higher than in healthy horses and horses with non-inflammatory disease (*P* < 0.0001), while concentration in healthy horses and horses with non-inflammatory disease did not differ significantly (*P* = 0.8). Overview of diagnoses/reason for hospitalization in the three groups of horses can be found in Additional file 2.

### Reference interval

The reference interval (RI) was determined in a diverse group of healthy horses. Age ranged from 1 day to 20 years (mean = 4.9 years), 22 breeds were represented. There were 30 geldings, 37 mares, and 36 stallions; gender was unknown in one horse (Additional file 3). The RI was determined (using the upper limit of the 90% confidence interval) to be up to 23.6 mg/L (Table 3) with 96/104 (92.3%) horses having SAA concentrations < 5 mg/L.

**Table 3:** Reference interval (and confidence intervals) determined in the VET-SAA assay

*n*	Median (mg/L)	Range (mg/L)	Reference interval (RI) (mg/L)	90% confidence interval of lower limit of RI (mg/L)	90% confidence interval of upper limit of RI (mg/L)
104	1.3	< 1.2–23.6	< 1.2–12.5	< 1.2	7.0–23.6

### Analyses of clinical and experimentally induced inflammation

**Fig. 5:**
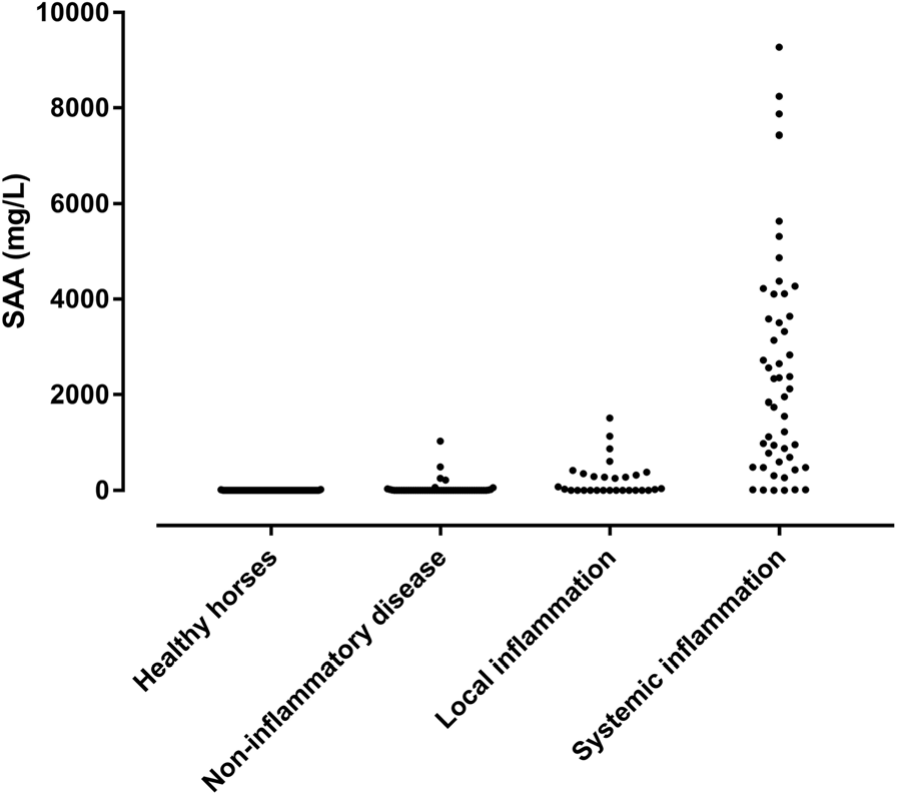
Serum concentrations of serum amyloid A (SAA) determined in healthy horses and horses with different inflammatory disease by means of an immunotubidometric assay developed for determination of SAA concentrations in horses (VET-SAA). Concentrations of SAA were significantly higher in horses with systemic inflammation than in the other three groups (*P* < 0.0001), while concentrations in healthy horses, horses with non-inflammatory diseases and horses suffering from local inflammation did not differ statistically significantly (*P* > 0.05)

Concentrations of SAA differed significantly between groups of horses with different inflammatory status (*P* < 0.0001). In healthy horses, average (range) SAA concentration was 2.0 (0–23.6) mg/L, in horses with non-inflammatory diseases it was 45.1 (0.2–1036) mg/L and in horses with local and systemic inflammation average (range) SAA concentrations were 238.7 (0.7–1517) and 2494 (4.3–9275) mg/L, respectively (Fig. 5). Concentrations of SAA were significantly higher in horses with systemic inflammation than in the other groups (*P* < 0.0001), while concentrations in the other three groups of horses did not differ.

**Fig. 6:**
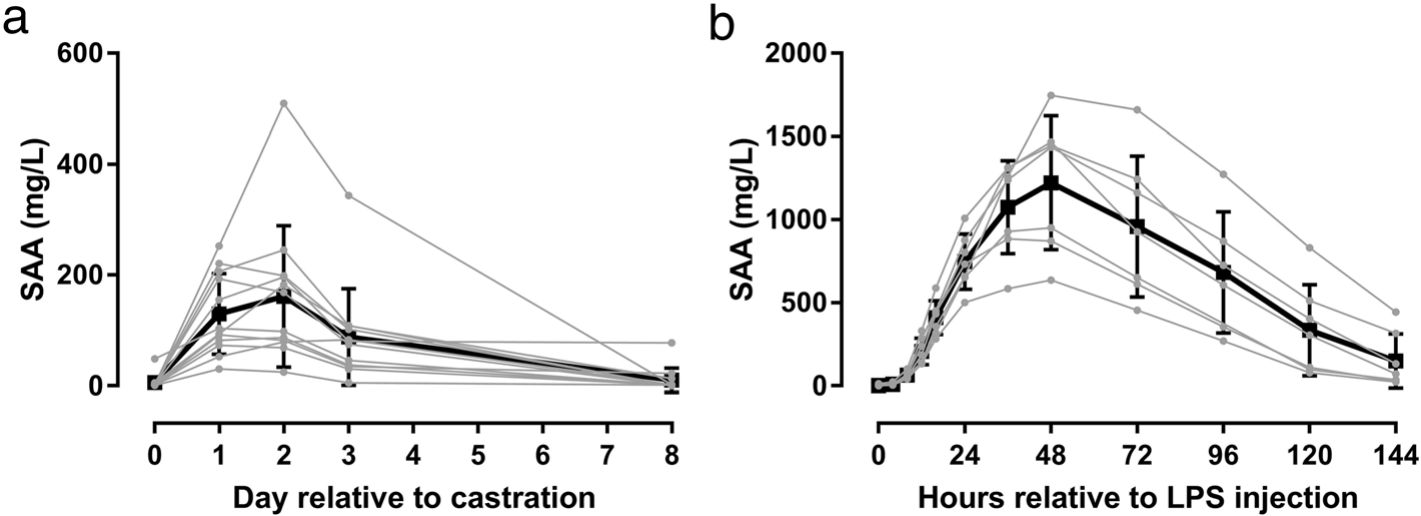
Serum concentrations of serum amyloid A (SAA) determined in horses undergoing castration and experimental systemic inflammation. Individual (grey lines) and average (black lines, error bars show standard deviations) serum amyloid A (SAA) concentrations in 12 horses before and 1, 2, 3 and 8 days after castration (**a**) and in 7 horses before and 4, 8, 12, 16, 24, 36, 48, 72, 96, 120 and 144 h after IV injection of lipopolysaccharide (LPS) (**b**). Serum concentrations of SAA were determined by means of an immunotubidometric assay developed for determination of SAA concentrations in horses (VET-SAA)

SAA concentrations increased significantly in horses with inflammation induced by castration (*P* = 0.0006) or experimental IV injection of lipopolysaccharide (LPS) (*P* < 0.0001) (Fig. 6). After castration, SAA levels were higher than pre-surgical levels on day 1 (*P* = 0.0005), 2 (*P* = 0.006), and 3 (*P* = 0.03); on day 8 concentrations had returned to pre-surgical levels. After systemic inflammation induced by IV injection of LPS, SAA concentrations were increased relative to pre-injection levels from 4 to 96 h.

## Discussion

The VET-SAA assay performed reliably. Repeatability was good, and acceptable accuracy (linearity under dilution) was observed in a very broad concentration range. In the most frequently encountered/relevant concentration range (0–3000 mg/L) the assay showed no signs of inaccuracy. When the entire concentration range (0–6159.7 mg/L) was analysed, slight signs of inaccuracy were detected (Table 2) consisting of a slight overestimation in the range of 3000–6000 mg/mL (Fig. 1). Considering the response pattern of SAA with fast and pronounced concentration increase in response to an inflammatory stimulus and a short half-life with resulting quick concentration decline after effective treatment [3], the observed minor inaccuracy is considered clinically insignificant, as the inaccuracy at no point prevents detection of increases or declines in SAA concentrations (Fig. 1).

Concentrations measured in the VET-SAA assay were significantly correlated with concentrations in the previously validated LZ SAA assay in the 0–3000 mg/L range. Beyond this range of concentrations, a proportional and systematic bias between the two assays was detected (Figs. 2 and 3); this was caused by the quantitative inability of the LZ SAA assay at higher concentrations. In the 0–3000 mg/L concentration range, SAA concentrations measured in the VET-SAA assay were significantly lower than in the LZ SAA assay; this proportional difference was, however, small (8%), which ensures a considerable agreement on clinical classification between the two assays and has the added benefit that clinicians will not have to get acquainted with another concentration range. Due to the heterologous calibration of the assay, the true concentration of equine SAA is unknown. No standard preparations of purified equine SAA are available, possibly due to the well-known difficulties in purifying SAA [17]. Consequently, in the VET-SAA, LZ SAA and other SAA assays used in equine medicine, calibration curves are based on recombinant human SAA, and concentrations are thus expressed as human equivalents. Between-assay variations in affinity of the antibodies towards the different isoforms [18] of the equine SAA protein may result in different concentration read-outs, as a higher affinity will translate into a higher SAA concentration read from the calibration curve. This may be the explanation for the two assays (both of which perform heterologous SAA assessment) yielding slightly different concentrations. Such slight difference in reactivity towards equine SAA and the human recombinant SAA calibrator could likely also be the source of the slight deviation from linearity under dilution described above, as this could result in lack of parallelism between the signals generated by the diluted sample and the calibration curve (a prerequisite for perfect linearity) .

There was no prozone effect in the assay. Prozone effect (also called hook effect) may occur when there is an excess of analyte and all antibodies in the assay are completely saturated. This is a highly undesirable feature, as it results in falsely low concentration outputs that goes unnoticed unless samples are analysed in several dilutions. Particularly when working with equine SAA, where markedly high concentrations are observed frequently [10, 19, 20], it is of great importance to ascertain that the assay does not have prozone issues. Within the concentration range of 0 to more than 6000 mg/L SAA the VET-SAA assay did not show prozone effect, similar to a previously validated multi-species SAA assay from the Eiken Chemical Co. [4]. The VET-SAA assay was able to measure SAA concentrations up to more than 9000 mg/L (Fig. 4). In the fairly rare occasions where SAA concentrations exceed 6000 mg/L, users should be aware that accuracy is unknown and further dilution of the sample may be advisable. To have a working range of 0 to 6000 mg/L SAA is exceptional for a clinically relevant, random-access assay and renders the assay particularly well suited for use in equine medicine.

SAA concentrations determined in the VET-SAA assay were not affected by haemolysis or lipaemia. Concentrations of the interfering molecules were similar to those used in a previous study, which showed no effect of these on SAA assessed in a commercially available ELISA [21]. Concentrations of haemoglobin and lipid were chosen to represent those observed in diseased horses, and SAA concentrations can thus be measured correctly in samples from horses with haemolytic disease or lipaemia.

As described in our review on assay validation [2], the purpose of assessing overlap performance (phase II of test validation) is to detect differences in analyte concentrations between healthy and clearly diseased individuals. This should be seen as a resource-sparing prelude to the resource-demanding phase III, in which clinical performance of the assay is evaluated. Our results were as expected: horses with moderate-severe inflammatory disease had significantly higher blood SAA concentrations than healthy horses and horses with non-inflammatory disease (Fig. 4). We expanded the simple phase II overlap assessment and showed that in horses with local inflammation SAA concentrations were not significantly different from those found in healthy horses and horses with non-inflammatory disease (Fig. 5). It has been noted in previous studies that local inflammation might not give rise to increased blood concentrations of SAA, potentially because of the walled-off nature of diseases such as Rhodococcus pneumonia or abscesses [8, 22].

The temporal concentration changes observed after a surgical trauma (castration) and experimental induction of systemic inflammation by IV injection of LPS (Fig. 6) were as expected and as shown in previous studies [23, 24]. These results demonstrate that the assay is fully capable of monitoring expected changes in SAA levels throughout relevant clinical phases of inflammation.

The upper limit of the 90% confidence interval of the RI was 23.9 mg/L, which would constitute the diagnostic cut-off when based solely on the data on healthy horses. Using this cut-off, the diagnostic specificity would by definition be 97.5%; it should, however, always be considered whether the distribution of pathological levels of the analyte may allow establishment of a clinical decision level that would increase the diagnostic sensitivity without compromising diagnostic sensitivity. If so, the clinical applicability of the test would be enhanced (by increasing the positive predictive value and thus ability to ‘rule-in’ presence of pathology) [25]. This is the case for SAA, as the diagnostic sensitivity of major acute phase proteins is provided by the large relative change in concentration in response to inflammation, not by adopting a cut-off level close to the RI with the purpose of identifying individuals with the slightest increases in serum SAA. Using a cut-off limit somewhat above the normal range can enhance the diagnostic specificity of equine SAA without seriously impairing diagnostic sensitivity [26], as most horses suffering from an inflammatory condition will have serum SAA levels that are greatly elevated above the upper limit of the RI. Thus, we propose that using a clinical decision level is more relevant than using the RI for differentiating healthy and diseased horses. Using the LZ SAA assay such a clinical decision limit of 30 mg/L was established at the Large Animal Teaching Hospital, University of Copenhagen, Denmark and has been used for more than 10 years. Considering the fact that the VET-SAA assay measures concentrations essentially similar to the LZ SAA assay, our current decision level of 30 mg/L may prove to remain relevant.

In conclusion, the VET-SAA assay measured SAA in equine serum with analytical performance acceptable for clinical purposes. It was able to detect the expected differences in SAA concentrations between healthy horses and horses with different inflammatory and non-inflammatory diseases as well as concentration changes occurring over time after induction of inflammation. With its reliability in an exceptional concentration range, it is particularly well-suited for the equine species, where SAA concentrations range from essentially 0 mg/L in healthy individuals to > 5.000–10.000 mg/L in horses suffering from severe systemic inflammation. The assay is automated, rapid, and applicable for random-access analysers, which facilitates use in routine diagnostic laboratories. Furthermore, since the VET-SAA is based on monoclonal antibodies, it may be associated with a strong long-term and inter-batch performance [14].

## Conclusions

The new immunoturbidometric assay (VET-SAA, Eiken Chemical Co., Japan) is unique in its ability to measure SAA concentrations with acceptable reliability over an extreme concentration range (up to > 6000 mg/L). This is relevant in the equine species, where SAA concentrations may reach very high concentrations. The detection limit is 1.16 mg/L, and the RI was determined to be up to 23.6 mg/L. The assay was able to differentiate horses with different inflammatory status and to monitor changes in inflammatory activity. Its characteristics (automated, rapid, based on monoclonal antibodies) confers a high applicability.

## Methods

### Serum amyloid A analyses

The VET-SAA assay (Eiken Chemical Co. Tokyo, Japan) is an immunoturbidometric assay using monoclonal rat anti-human SAA1 antibody. Analyses were performed using an automated analyser (Advia 1800, Siemens Healthineers, Ballerup, Denmark) according to the manufacturer’s instructions. These instructions entail a 1:4 pre-analysis dilution of each sample and, when SAA concentration in the sample exceeds 200 mg/L, a further 1:14 reflex dilution is performed as an integral part of the assay conditions on the automated analyser. The calibration curve was made using the calibrator (human recombinant SAA) supplied with the kit.

For method comparison, samples were analysed using the previously validated immunoturbidometric LZ SAA assay (Eiken Chemical Co. Tokyo, Japan), which is based on polyclonal rabbit anti-human SAA antibody and monoclonal rat anti-human SAA1 antibody and uses human recombinant SAA as a calibrator [13]. This assay was used according to the manufacturer’s instruction (which entails a 1:4 pre-analysis dilution of samples) with the exception that all samples containing SAA concentrations > 300 mg/L underwent a reflex dilution of 1:5 and were re-analysed automatically, as previous studies had demonstrated the need for sample dilution to expand the working range of the assay [12, 13]. No manual sample dilution was performed.

### Assay characteristics

Precision was assessed by intra- (same day) and interassay (non-consecutive days) CV from the mean and SD of 10 replicate determinations of three serum pools containing low, intermediate and high concentrations of SAA (Table 1). Pools were obtained by mixing serum from at least 5 different horses. To avoid effect of repeated thawing and freezing, pools used for the determination of inter-assay variation were aliquotted and stored at − 80 °C until use. Only aliquots needed for each analytical run were thawed. Inaccuracy was investigated by evaluating linearity under dilution. This was performed as serial dilutions of a serum pool containing a very high concentration of SAA (6159.7 mg/L), which was diluted to obtain sample volume percentages of 0, 1, 2, 3, 4, 5, 6, 7, 8, 9, 10, 15, 20, 25, 30, 35, 40, 45, 50, 55, 60, 65, 70, 75, 80, 85, 90, 95 and 100%.

The DL was determined by 20 replicate determinations of ionized water.

Interference of haemolysis and lipaemia was tested. A haemolytic solution was produced by the osmotic shock procedure described by the CLSI guidelines (Guideline EP07, https://clsi.org/standards/products/method-evaluation/documents/ep07/). In brief, EDTA-stabilized equine blood was centrifuged, plasma was discarded and the cell pellet was washed three times using 0.9% NaCl. Erythrocytes were lysed by adding distilled water and freezing the sample overnight. After thawing and a single washing procedure to remove cellular debris, a solution containing approximately 100 g/L haemoglobin was obtained. A commercial fat emulsion solution (Intralipid 20% emulsion, Sigma Aldrich, VWR International, Søborg, Denmark) was acquired. The haemoglobin and lipid solutions were added to two pools of equine serum containing approximately 110 mg/L and 560 mg/L SAA in different concentrations (total concentration of haemoglobin = 0.625 g/L, 2.5 g/L or 10 g/L; total concentrations of lipids = 0.31 g/L, 1.25 g/L or 5 g/L) and SAA concentrations assessed by the VET-SAA assay 3 times (Additional file 1). Acceptance criterion was set at −/+ 10%.

### Animals and samples

To assess overlap performance of the assay for detection of systemic inflammation, samples from healthy horses (*n* = 18), horses suffering from non-inflammatory disease (*n* = 17) and horses suffering from diseases characterized by moderate to severe systemic inflammation (*n* = 15) were included. To compare SAA levels in horses with different inflammatory states, an additional 86 healthy horses, 51 horses with non-inflammatory disease and 50 horses with systemic inflammatory disease of varying severity were included along with 29 horses suffering from localized inflammation. The patient samples originated from equine patients presented at the Large Animal Teaching Hospital at University of Copenhagen, Denmark. All patients underwent clinical examination, routine haematology and serum biochemistry and additional diagnostic procedures such as diagnostic imaging, synoviocentesis, endoscopic procedures, and/or diagnostic analgesia to achieve a final diagnosis. Horses with non-inflammatory disease were diagnosed with non-strangulating intestinal conditions (*n* = 14; small and large intestinal obstipations, nephro-splenic large colon entrapment, right dorsal displacement of the large colon), musculoskeletal conditions (*n* = 15; navicular disease, flexural deformity, osteoarthrosis, upward fixation of the patella, radial nerve paralysis, kissing spines, unrideability), cardiac conditions (*n* = 6; mitral or aortic valve insufficiency), dental disease (*n* = 3; equine odontoclastic tooth resorption and hypercementosis), upper airway diseases (*n* = 2; laryngeal hemiplegia, pharyngeal collapse), metabolic diseases (*n* = 5; hyperlipidemia, pituitary pars intermedia dysfunction), and miscellaneous conditions (n = 6; umbilical hernia, behavioural issues, retinal detachment, blocked tear canals). Horses with systemic inflammation were diagnosed with orthopedic infections (*n* = 16; lymphangitis/cellulitis, wound infections, septic synovitis, hoof abscess), gastrointestinal and intraabdominal infections (*n* = 18; colitis, enteritis, peritonitis), airway infections (*n* = 5; pneumonia, pleuropneumonia), pyrexia (*n* = 5), and miscellaneous conditions (*n* = 6; bladder rupture, septicemia, neoplasia, retained placenta, thymitis, neuritis). Horses with localized inflammation were diagnosed with abscesses/fistulas (*n* = 12), strangles/lymphadenopathy (*n* = 4), rectal tears (*n* = 3), septic funiculitis/scirrhous cord (*n* = 4), phlebitis (*n* = 1), sinusitis (*n* = 3), and guttural pouch infections (*n* = 2).

The resulting 104 (18 + 86) samples from healthy horses were used for determining a population-based RI of SAA.

Two sets of sequential serum samples were available from two previous studies involving 12 horses undergoing castration (unpublished data) and 7 horses subjected to IV injection of LPS resulting in systemic inflammation [23]. In horses undergoing castration, samples were obtained before and 1, 2, 3 and 8 days after the surgical procedure. The castrated horses were clinically healthy before surgery and had a normal postoperative recovery. In horses with experimental LPS-induced inflammation, samples were obtained at 0, 4, 8, 12, 16, 24, 36, 48, 72, 96, 120 and 144 h relative to the injection.

### Statistical analyses

Arithmetic means, standard deviations, medians, intra- and inter-assay CVs were estimated using routine descriptive statistical procedures. The DL (*P* < 0.01) was estimated as the mean + 3 SD of SAA determination of blank samples. Linearity under dilution was investigated by linear regression analysis. Runs test was performed to determine whether data deviated significantly from the applied model. The effect of different concentrations of haemoglobin or lipid on measured SAA concentrations were examined by calculating the bias percentage as described by Kjelgaard-Hansen and Jensen [27] (Additional file 1). Method comparison of the two assays was performed by Deming regression analysis and visualized in Bland-Altman plots. Calculation of RI and 90% confidence interval was performed with a dedicated software (Reference Value Advisor, http://www.biostat.envt.fr/reference-value-advisor/) using nonparametric methods due to the skewed distribution of native data (determined by the D’Agostino-Pearson omnibus test) as described by Geffre et al. [28]. Overlap performance was assessed by comparing groups (healthy, non-inflammatory disease, inflammatory disease) using the Kruskal–Wallis test; when significant results were obtained, Dunn’s multiple comparison test was performed. Differences between groups with different inflammatory status (healthy, non-inflammatory, local inflammation, systemic inflammation) were analysed with one-way ANOVA and the post hoc test Tukey’s multiple comparisons test. Changes in SAA levels in castrated horses and horses with LPS-induced inflammation were evaluated using the repeated measures ANOVA and Tukey’s multiple comparisons post hoc test. A level of significance of 0.05 was used unless otherwise stated. Statistics were performed using a commercial package (Graph PadPrism 8.00 for windows, GraphPad Software, La Jolla, California, USA).

## Supplementary information

**Additional file 1.** Interference testing, methods and results.

**Additional file 2.** Overview of diagnoses/reason for hospitalization in the three groups of horses used to assess overlap performance.

**Additional file 3.** Overview of healthy horses used for calculating reference interval.

## Data Availability

The datasets used and/or analysed during the current study are available from the corresponding author on reasonable request.
