# Supplementary material for: Validation of an equine serum amyloid A assay with an unusually broad working range
Source: BMC Vet Res. 2019 Dec 19;15:462. doi: 10.1186/s12917-019-2211-3 (PMC6923866; doi:10.1186/s12917-019-2211-3)
Supplement: Supplementary file 1 — Additional file 1. Interference testing, methods and results. [file 12917_2019_2211_MOESM1_ESM.docx]

# Interference examination of VET-SAA in equine serum samples

## Generation of pools

Low and high pool of equine SAA was generated using serum from 24 horses (12 horses in each pool). resulting in a low pool with the SAA concentration of approximately 110 mg/L and an intermediate pool with the SAA concentration of approximately 560mg/L.

## Production of hemoglobin

1. Eight mL of EDTA-stabilized equine blood is centrifuged at 3300 x *g* in 5 minutes
2. Plasma is discarded and the cell pellet washed three times using 0.9 % NaCl
3. Erythrocytes are lysed by adding distilled water and the sample is frozen overnight
4. After thawing and a single washing procedure to remove cellular debris, a solution containing approximately 100 g/L is produced. This was confirmed by measuring the content using an Advia 2120i, Siemens, Germany.

## Interference with hemolysis

Three different concentrations of hemoglobin (0.625 g/L, 2. 5g/L, and 10 g/L) diluted in demineralized water reflecting low, intermediate and high hemolysis (CLSI E07) as well as a control (0 g/L) was measured in triplicates (table 1). An volume of demineralized water similar to that of the hemoglobin solution was added to the control sample to control for dilution effects.

## Acquisition of lipids

As the optimal lipid formulation is not available for mimicking lipemia, it is recommended to use a commercial product).

## Interference with lipemia

Three different concentrations of the lipid formulation (Intralipid 20% emulsion (Sigma Aldrich, VWR International, Søborg, Denmark) (0.625 g/L, 2.5 g/L, and 10 g/L) diluted in demineralized water reflecting low, intermediate and high lipemia (CLSI E07) as well as a control (0 g/L) was measured in triplicates (table 2). An volume of demineralized water similar to that of the lipid solution was added to the control sample to control for dilution effects.

## Acceptance criteria for the interference testing

The performance goal is that the difference (bias percentage) between the control sample and the samples with added either hemoglobin or lipid formulation are -/+ 10%. This is calculated by the formula ((control sample – measured sample with interfering substance)/control sample) * 100.

## Results

All bias percentages were within -/+10% as seen in tables 1 and 2.

| Table S1: Spike in with different hemoglobin concentrations performed in equine serum pools with low and intermediate concentrations of serum amyloid A. | | | | | | |
| --- | --- | --- | --- | --- | --- | --- |
|  | Hemoglobin (g/L) | Equine serum pool (Low/Intermed.) | Mean  SAA (mg/L) | Standard deviation | Bias (mg/L) | Bias % |
| Control | 0 | L | 114.4 | 8.0 | - | - |
| Low hemoglobin | 0.625 | L | 115.7 | 5.8 | 1.3 | 1.1% |
| Intermed. hemoglobin | 2.5 | L | 116.5 | 6.2 | 2.1 | 1.8% |
| High hemoglobin | 10 | L | 118.0 | 1.4 | 3.6 | 3.1% |
| Control | 0 | I | 577.6 | 9.7 | - | - |
| Low hemoglobin | 0.625 | I | 584.3 | 49.5 | 6.7 | 1.2% |
| Intermed. hemoglobin | 2.5 | I | 589.0 | 17.3 | 11.4 | 2.0% |
| High hemoglobin | 10 | I | 629.6 | 11.7 | 52 | 9.0% |

| Table S2: Spike in with different lipid concentrations performed in equine serum pools with low and intermediate concentrations of serum amyloid A | | | | | | | |
| --- | --- | --- | --- | --- | --- | --- | --- |
|  | Lipid (g/L) | Equine serum pool (Low/Intermed.) | Mean  SAA (mg/L) | Standard deviation | Triglycerides  (mmol/L) | Bias (mg/L) | Bias % |
| Control | 0 | L | 141.4 | 9.7 | 0.5 | - | - |
| Low lipid | 0.31 | L | 139.8 | 2.9 | 1.3 | -1.6 | -1.1% |
| Intermed. lipid | 1.125 | L | 134.0 | 8.3 | 3.3 | -7.4 | -5.3% |
| High lipid | 5 | L | 139.2 | 9.5 | 12.3 | -2.2 | -1.6% |
| Control | 0 | I | 672.1 | 49.6 | 0.6 | - | - |
| Low lipid | 0.31 | I | 610.7 | 10.4 | 1.3 | -61.4 | -9.1% |
| Intermed. lipid | 1.125 | I | 716.1 | 86.5 | 3.2 | 44.0 | 7.2% |
| High lipid | 5 | I | 687.6 | 72.0 | 12.9 | 15.5 | 2.2% |
